# The Theoretical Lineage and Evolutionary Logic of Research on the Environmental Behavior of Family Firms: A Literature Review

**DOI:** 10.3390/ijerph20064768

**Published:** 2023-03-08

**Authors:** Limin Geng, Xueyuan Lu, Can Zhang

**Affiliations:** College of Economics and Management, Nanjing Forestry University, Nanjing 210037, China

**Keywords:** family firms, corporate environmental behavior, heterogeneity, SEW

## Abstract

Family firms research is becoming one of the most important and promising areas for theoretical innovation in management practice. Corporate environmental behavior has attracted widespread academic attention, but the research on the environmental behavior of family firms is obviously insufficient, and the relevant research results are still in a fragmented state. In this paper, we review and summarize the existing research on the environmental behavior of family firms from three aspects: the research dimensions, the influencing factors, and the influencing effects, and try to sort out the theoretical lineage and evolutionary logic of the environmental behavior of family firms. From the existing research results, the research on the influencing factors and effects of family firms’ environmental behavior is at the stage of strife, and there is a lack of in-depth and systematic research on the mechanisms affecting the environmental behavior of family firms and the changes of their effects. In the future, we can explore how to apply or integrate multiple theories simultaneously for complementary explanations, so as to provide a reference for the government to formulate targeted policies to stimulate and regulate the environmental behaviors of family firms.

## 1. Introduction

With accelerated industrialization and the deterioration of the ecological environment worldwide, combating climate change and protecting the ecological environment has become the common goal of all countries. Enterprises are both economic developers and environmental polluters. Therefore, while focusing on environmental policy research, scholars have begun to study the self-interested responses of enterprises under multiple environmental pressures from the perspective of enterprises, and research on corporate environmental behavior has increasingly become a hot issue in the academic field [1]. Corporate environmental behavior refers to the adjustment measures taken by enterprises in the face of external environmental pressures in their production and operation. Due to the wide involvement of stakeholders, the factors, mechanisms, and effects of corporate environmental behavior are more complex and have more important strategic significance.

Family firms are the oldest and most important organizational form in the history of human business, as well as modern organizations that are vigorous and influential in the contemporary economy and society. Both in developed economies such as the United States and European countries, as well as in developing economies, the proportion of family firms and the contribution they make to the economy is very significant [2,3,4]. Family firms have unique behavioral characteristics due to the interaction and dynamics of family and firm systems [2,5], and family firm research is becoming one of the most important and promising areas for theoretical innovation in management practice, attracting more and more management, economics, sociology, and other disciplines’ attention [6]. The unique behavioral characteristics of family firms lead to certain differences in their environmental awareness and behavior compared to general enterprises. Organizational identity theory, organizational reputation theory, and agency theory have all become important theoretical foundations to explain the differences in the fulfillment of environmental responsibility of family firms [7,8]. Family firms, as complex systems formed by family involvement in business, will be significantly affected by family involvement factors, and the prior research literature has produced different findings based on different theoretical analysis frameworks.

In recent years, many scholars in important international journals have conducted theoretical and literature reviews on family firms, which have sorted out the theoretical and empirical studies on family firms from different perspectives [9,10]. They also have given the historical lineage, current status of research, and outlook of family firm research, which have contributed to the systematization of family firm-related research. However, the research on the environmental behavior of family firms is still in its infancy, both in terms of theoretical construction and the knowledge system of the discipline. Scholars are still developing their concepts, connotations, and theoretical foundations, and there is a lack of systematic literature on the environmental behavior of family firms, which is regrettable for the further development of family firm research. It is against this background that this paper aims to provide a theoretical overview of the theoretical development of the environmental behavior of family firms, focus on the factors and effects of the environmental behavior of family firms, and discuss the research progress rooted in the study of the environmental behavior of family firms in recent years, so as to provide a theoretical overview for the subsequent research of scholars and explore the evolutionary logic of the study of the environmental behavior of family firms.

The research idea of this paper is as follows: review and summarize the existing literature and sort out the theoretical lineage of family firms’ environmental behavior by dividing the dimensions of family firms’ environmental behavior based on distinguishing the heterogeneity of general firms and family firms. Firstly, we compared and summarized the application and development of existing theories in the environmental behavior of family firms from the dimensions of environmental responsibility, economics, and corporate strategy. Secondly, this paper discusses the influencing factors and effects of family firm environmental behavior and the research progress of family firm environmental behavior under different theoretical backgrounds based on distinguishing homogeneity and heterogeneity. Finally, based on the cross-research between family firms and related fields, the development direction of family firm environmental behavior under the background of the increasing importance of environmental health and digitalization is discussed.

## 2. Research Dimensions of the Environmental Behavior of Family Firms

### 2.1. Definition of Family Firms

How to define a family firm is the starting point of family firm research, and the history of family firm research is basically the process of deepening the understanding of the family firm’s concept, connotation, and characteristics [11]. Allen and Panian (1982) first defined family firms [12], and they consider it a family firm as long as a descendant member and his relatives own or control at least 5% of a company’s voting stock and are represented on the board. McConaughy et al. (1998) considered any firm run by the founders or founding family members as a family firm [13], and subsequent studies have considered firms in which the founding family or founding individuals own a portion of the firm’s shares or serve on the board of directors as family firms [2,14,15]. Astrachan et al. (2002) developed a scale (power, experience, and culture) to comprehensively evaluate the influence of family on the firm, using continuous variables to measure the characteristics of the firm [16]. Later studies generally believed that family ownership and control power are important characteristics of family firms, which make them different from other enterprises. Hence, they tend to pay more attention to their specific family goals and be more willing to retain the company’s management discretion and control power [4,17,18,19,20,21]. In studies addressing the environmental behavior of family firms, scholars have generally adopted Allen and Panian’s definition [22,23] or a stricter criterion that considers firms with at least 10% of shares owned or controlled by family members as family firms [24]. It is worth noticing that, during the empirical analysis, most scholars tend to adopt multiple criteria to define family firms and thus ensure the robustness of the empirical results.

### 2.2. Corporate Environmental Behavior

Different scholars or institutions have different definitions of corporate environmental behavior. The U.S. National Environmental Protection Agency defined corporate environmental behavior as actions taken by firms that are used to improve environmental performances or comply with environmental laws and regulations. Sarkar (2006) defined corporate environmental behavior as a set of strategies involving firms managing the relationship between business operations and the environment, either as a response to external pressures or as proactive initiatives to mitigate environmental hazards [25]. Similar concepts include corporate pro-environmental behavior [26], which refers to behaviors that mitigate environmental hazards or benefit the environment, and corporate green behavior [27], which refers to a variety of measures taken by firms to respond to environmental issues, including proactive waste disposal, energy consumption reduction, waste emission reduction, and the use of cleaner production technologies. Although these concepts differ in terms of designation, the connotations all emphasize the enterprises’ active participation in ecological and environmental management and protection or their commitment to solving environmental problems, etc. The study of the environmental behavior of family firms is a subdivision of the study of corporate environmental behavior. In the study of the environmental behavior of family firms, the concept of environmental behavior is more diverse, such as proactive environmental strategy (PES), environmental sustainability orientation (ESO), and environmental sustainability (ES), as proxies for corporate environmental behavior [28].

### 2.3. Research Dimensions of the Environmental Behavior of Family Firms

Relevant research on corporate environmental behavior involves many topics, and the hierarchical relationship is extremely complex. This is mainly because many internal and external factors, as well as the interaction between these factors, can affect corporate environmental behavior and lead to the diversification of corporate environmental behavior. In addition to the existing research of corporate environmental behavior, the study of family firm environmental behavior, which is mainly carried out from the dimensions of environmental responsibility, economics, corporate strategy, provides a new perspective (see Table 1).

#### 2.3.1. Dimension of Environmental Responsibility

The research of family firm environmental responsibility is mostly based on the theory of social emotional wealth (SEW). Due to family’s concern about image and reputation and the desire to protect family assets, family firms have a more sense of social responsibility [29] and are more inclined to build a good image for the firm and family by fulfilling social environmental responsibilities, helping the firm to gain more attention from other stakeholders, and promoting their continuation and sustainable development. From the perspective of the motivation of family firms to maintain social emotional wealth, the more involved the family firms are, the more actively they will fulfill their social and environmental responsibilities, and the better their ESG rating performance will be [30,31]. However, some studies believe that if family members have low control power, other shareholders may be more inclined to pay attention to the short-term financial performance of the firm and have less willingness to undertake social responsibilities [32]. Liu et al. (2017) studied the relationship between corporate social responsibility (CSR) activities of family firms and earnings management, and found that family firms engaged in less accrual-based earnings management, and there was no difference between them and non-family firms in terms of CSR activities and actual earnings management [33]. However, some studies believed that family firms exhibit more socially responsible behaviors in order to protect their social emotional wealth, which is negatively correlated with earnings management practices [34].

The conclusions of research on environmental practices, environmental information disclosure and environmental reporting of family firms are not completely consistent. Doluca et al. (2018) studied the environmental behavior of family firms in terms of environment-related activities, innovation, and performance, and found that family firms lagged slightly behind non-family firms [35]. However, research based on the combination of social emotional wealth (SEW) theory and institutional theory found that there was a positive relationship between family firms and environment-friendly practices (EFP), and family ownership was positively correlated with the tendency to participate in pollution prevention [36]. The disclosure of environmental performance information may weaken the control of family firms over their enterprise group companies, but it also generates reputation. Therefore, the impact of family control on the disclosure of environmental information is neutral, and will be affected by the control level of family ownership and the comprehensive impact of managers [37]. Family firms provide less environmental information than non-family firms, but this impact will be weakened in the life cycle of family firms [38]. In addition, relevant studies on corporate earnings management (EM) and CSR found that the stage of the industry life cycle affects the relationship between EM and CSR [39]. Compared with non-family firms, when earning management is reduced, family firms are more inclined to divert people’s attention from these practices through CSR disclosure [40], and ESG performance has a significant moderating effect on earnings management [41].

#### 2.3.2. Dimension of Economics

The relevant research based on the economic dimension no longer regards the implementation of positive environmental behavior as the moral responsibility of enterprises, but as the functional choice of enterprises, and studies the influence of internal and external factors on the corporate environmental behavior, as well as the environmental behavior response of enterprises under the influence of environmental regulations and other policies. Environmental behavior based on family firms mainly focuses on environmental regulation and green innovation efficiency.

Environmental regulation can be divided into market-based environmental regulation and command-based environmental regulation. The impact of these two forms of environmental regulation on the environmental behavior of family firms is not the same. The green innovation of family firms under environmental regulation is essentially a trade-off between emotional benefits and emotional costs, which is manifested as reputation incentives and risk avoidance. Command-based environmental regulations can promote the green innovation efficiency of family firms, while market-based environmental regulations can inhibit it [42]. The intervention of family ownership strengthens the positive impact of command-based environmental regulation on green innovation efficiency, while the intervention of family management rights strengthens the negative impact of market-based environmental regulation on green innovation efficiency. With the involvement of family control and management, the incentive motivation and risk avoidance level of reputation are amplified [43]. In order to maximize the utility of family members, family firms tend to avoid more risks and ultimately change the efficiency of green innovative family firms and gain sustainable competitive advantages. In addition, the inheritance and environmental investment of family firms may be regulated by the government and the market [17,44].

#### 2.3.3. Dimension of Strategic Management

The relevant research based on the strategic dimension mainly focuses on the choice of corporate environmental behavior and the construction of corporate competitive advantage under the competitive advantage, which is the specific application of Porter’s “competitive strategy” thought in the environmental field. According to the Porter hypothesis, properly designed environmental regulations can promote enterprises to increase R&D, and enterprises that take the lead in environmental innovation can gain the “first mover” advantage [45]. The Porter hypothesis has deepened the research on corporate environmental behavior. Scholars start from the realization path of the Porter hypothesis and build an empirical model to study the realization conditions of “win–win” economic performance and environmental performance of enterprises. The research perspectives of family firms on economic performance and environmental performance are diverse and fruitful. See Section 4.2 for details.

The research on competitive advantage of family firms through environmental behavior mainly focuses on green strategy and long-term oriented strategy. The government’s environmental regulation and the market’s green consumption demand have put forward new requirements for the green development of family firms [46]. Especially for those family firms in the leading position in the industry, stakeholders such as government departments and consumers have a wide range of concerns about their green performance or investment, forming a dual system and public opinion pressure, thus driving the family firms that have become industry leaders to implement green strategies. Scholars generally believe that whether there is a long-term orientation is an important sign to distinguish family firms from non-family firms. Family firms with the intention of cross-generation inheritance will pay more attention to the future orientation and adhere to the long-term orientation strategy, which is also a key driving force for the choice of green strategy and an important source of competitive advantage [17].

## 3. Influencing Factors of Family Firm Environmental Behavior

The study of factors influencing the environmental behavior of family firms is a branch derived from the study of factors influencing the environmental behavior of general enterprises. The findings on the factors influencing the environmental behavior of general enterprises may also apply to family firms, but family firms also show different behavioral preferences in the fulfillment of their environmental responsibilities. The research on the influencing factors of the environmental behavior of family firms can be divided into two directions: one is to consider family firms as homogeneous and study the differences in environmental behavior between family firms and non-family firms, and the other is to consider the heterogeneity of family firms and seek the influencing factors of environmental behavior differences within family firms (see Table 2).

### 3.1. Homogeneity Perspective

Studies that consider family firms as homogeneous enterprises tend to dichotomize family and non-family firms, using “whether they are family firms”, “family ownership”, or “family involvement” as independent variables to find out whether family characteristics are conducive to the environmental behavior of firms. Different scholars in studies conducted with a homogeneity perspective have reached different or even opposite conclusions. Many of the scholars who found that the environmental behavior of family firms is better than that of non-family firms tend to adopt socioemotional wealth (SEW) theory to explain family environmental behavior, while some scholars have found that familiality has a negative effect on corporate environmental behavior based on SEW dark side theory. In recent years, studies have also begun to use agency theory to explain family firm environmental behavior and have reached strong explanatory conclusions.

Family firms often deviate from their own economic goals to pursue non-economic goals. Some scholars introduce concepts such as sentimental value, property attachment, altruism, and family values to explain this phenomenon. However, since each concept can only reveal a certain aspect of family firms’ pursuit of non-economic goals, relevant studies are scattered and lack of systematism. Based on this, Gomez-Mejia et al. (2007) proposed the concept of SEW on the basis of integrating previous research results to comprehensively investigate the non-economic impact of family firms [18]. SEW refers to the non-economic benefits that the family obtains from the family firms by virtue of its identity as owner, decision-maker ,and manager, specifically including the ability to exercise power, meet the needs of belonging, emotion and kinship, maintain family values within the firm for a long time, maintain family control, preserve family social capital, perform family obligations based on blood relationship, and treat family members with altruism [18]. SEW theory provides a new perspective for family firms research and can explain many family firms’ problems that cannot be explained by economic theories.

#### 3.1.1. SEW Theory and the Environmental Behavior of Family Firms

SEW theory is commonly used in management research and has been used to explain the differences that exist between family and non-family firms [18,47]. SEW theory assumes that family members manage firms to maintain and increase the social and economic benefits that the family receives from its participation in the firm. Therefore, family members may forego short-term benefits or put the firm’s economy and finances at risk in order to protect SEW when managing the firm [18]. The study of SEW for factors influencing the environmental behavior of family firms began with Berrone et al., who divided SEW into five dimensions: family control and influence, family members’ identification with the business, close social solidarity, family members’ emotional attachment and intergenerational inheritance intention [22]. Members of family firms have a deeper identification with the firm, and are more inclined to protect the family’s reputation and image. As a result, they tend to have closer social ties at the firm’s location, and so will be more inclined to increase the firm’s pro-environmental behavior [22].

SEW theory explains the promoting effect of family nature on corporate environmental behavior, which has had a profound influence on the research in this field. Since then, research on the environmental behavior of family firms from the dichotomous perspective of “family” and “corporate” has found that SEW goals reflect the family nature of family firms, while economic wealth goals and risk awareness reflect the corporate nature of family firms, and the two opposing goals together influence the environmental behavior of family firms [35]. The intergenerational commitment of SEW theory also influences the environmental behavior of family firms. The presence of family ownership facilitates the creation of a firm’s LTO (long-term orientation), in which firms tend to take environmental performance and environmental goals into account. Therefore, the positive effect of family ownership on corporate environmental behavior is mediated by LTO and family commitment [28]. In addition, compared to non-family firms, family firms will have a stronger propensity for green innovation driven by internal motivation for the preservation of extended socioemotional wealth and external institutional pressure [48], and the degree of family involvement can enhance the ESG (Environment, Social and Governance) rating performance of firms [49]. The relationship between ESG disclosure and earnings management is influenced by the firm’s family or non-family status [50]. Cross-cultural research has found that social emotional wealth (SEW) affects the environmental and social strategies (ESS) of family firms, and the performance of family firms varies under different cultural backgrounds, but relatively young family firms are more likely to adopt ESS, suggesting that intergenerational dynamics and selectivity theory may play a role [51].

#### 3.1.2. SEW Dark Side Theory and the Environmental Behavior of Family Firms

Different from the study of Berrone et al. (2010) [22], Kellermanns et al. (2012) believed that SEW could be seen as a double-edged sword, showing a bright or dark side, and negatively valenced SEW will lead to family-centered behaviors, which will have a negative impact on PSE (positive stakeholder engagement) and conduct harmful to stakeholders. Family firms that tend to pursue non-economic goals may lead to nepotism and the use of company resources for private gain [52]. Owners and leaders of family firms may use company assets to reward incompetent offspring, leading to distortions in the distribution of social resources, where some family members may benefit while other stakeholders are treated unfairly [53,54].

Corporate image maintenance and a high level of family involvement can have an impact on the environmental behavior of family firms. Family firms in polluting industries are more likely to fulfill their declared environmental commitments due to their desire to maintain a good family image. At the same time, family firms are less likely to invest in environmental protection due to goals to maintain the financial sustainability and pass on family wealth, and instead view investments in environmental sustainability as a net cost [55]. In addition, Samara et al. (2018) also analyzed from the bright and dark side perspective of SEW theory and concluded that absolute family ownership may reduce incentives and limit opportunities for family firms to improve their environmental and social performance. While the high level of family involvement in management may bring professionalism, internal family conflicts may also bring chaos to the management of the family firms, which will eventually reduce the resources available for improving the environmental and social performance of the family firms [56]. Zhou, and Zhao (2017) argued that Chinese family firms place more emphasis on the protection of SEW centered on family control [57], and decision-makers in family firms will prefer to invest limited resources in the development of the firm rather than environmental protection when making decisions, and there is a significant negative impact of family ownership and management involvement on the fulfillment of corporate environmental responsibility. In terms of environmental information disclosure, Arena and Michelon (2018) found that family firms provide less environmental information than non-family firms using a sample of companies listed on the Milan Stock Exchange and this effect diminishes over the life cycle of family firms [38].

#### 3.1.3. Agency Theory and the Environmental Behavior of Family Firms

In recent years, agency theory has also been applied to the study of factors influencing corporate environmental behavior, but the conclusions are also different. The traditional agency perspective assumes that the principal-agent costs of owner-operated enterprises (such as family firms) are low [58], but subsequent relevant studies show that family firms have significant principal-agent costs and will affect their environmental behavior. Family members try to seek private interests from the enterprise [59,60], such as nepotism, favoritism, and engagement [54,61], which may lead to lower enthusiasm and productivity of employees in environmental activities [62]. The inconsistency between the interests and goals of the family majority shareholder in family firms and those of minority or other majority shareholders leads to an increased likelihood of compromising the interests of non-majority shareholders [63], and the owner family’s pursuit of goals other than economic wealth will affect its environmental behavior [18]. Analysis of a sample of 2345 Chinese firms using agency and institutional theory found that family ownership of firms has a negative impact on pollution control of firms, leading to worse environmental behavior performance of family firms compared to non-family firms, but this is mitigated by having the founder as CEO [24]. In addition, other scholars have used methods such as meta-analysis from an agency perspective and concluded that family firms have worse environmental behavior than non-family firms [64,65]. Moreover, close shareholding and family ownership are negatively correlated with ESG performance [66]. However, Borralho (2022) used data from listed companies in France and Spain to investigate how each component of environmental, social and governance (ESG) disclosure individually affects the earnings management of family and non-family firms and found that the relationship between ESG disclosure and earnings management is influenced by family or non-family status [50].

It is noteworthy that studies focusing on environmental investment activities have reached different conclusions. Major shareholders may not necessarily bring loss of interests to minority shareholders when investing in environmental activities; that is to say, the second type of agency problem does not occur in the environmental behavior of family firms. Shareholders respond more positively to companies with higher environmental performance ratings, and the effect is stronger in family-owned firms (Cordeiro et al. 2021) [23]. Family firms are more responsible to shareholders than non-family firms when it comes to environmental investments. When shareholder and social interests are aligned—that is, when mitigating environmental issues that may harm society and increase the risk of the firm—family firms are at least as protective of shareholder interests as non-family firms; when shareholder and social interests diverge—that is, when making environmental investments that may benefit society but disadvantage shareholders—family firms prioritize the protection of shareholder interests [67]. In conclusion, from an agency theory perspective, most studies have argued that the family nature of family firms leads to the second type of agency problem thus making the environmental behavior of firms worse compared to non-family firms. However, the agency theory perspective only compares the environmental behavior of family firms with that of non-family firms from an internal behavioral theoretical level, and there is a relative lack of consideration of the external environment.

#### 3.1.4. Other Homogeneity Studies

There are few studies on the environmental behavior of family firms from other perspectives. Based on the resource-based view and the theory of planned behavior, it is found that family influence has a positive impact on the participation of SMEs (Small and Medium-sized Enterprises) in environmental management practices [68]. Dekker and Hasso (2016) argued that when assessing the environmental behavior of families, more attention should be paid to the family’s emphasis on the environment in the overall business performance assessment and that those family firms with strong social embeddedness in the local community come to be more concerned about environmental performance than non-family firms [69]. A study based on data from the 10th National Private Enterprise Survey in China found that family involvement has a significant positive effect on environmental behavior, but family involvement is significantly negatively related to the willingness to fulfill corporate environmental governance; the institutional environment is a key macro factor affecting the performance of voluntary family social responsibility, and the positive effect of family involvement on corporate environmental behavior is stronger in a more market-oriented environment [70]. In addition, Dangelico et al. (2019) used a sample of 14 Italian small firms engaged in the agri-food industry and found that family and non-family firms were particularly similar in aspects such as technological innovation, but weaker than non-family firms in terms of firm motivation and performance of environmental behaviors such as green innovation [71]. Other studies have examined the impact of philanthropic behavior, public incentives, environmental regulation, and institutional characteristics on the environmental behavior of family firms based on different samples and reached different research conclusions [24,72,73].

### 3.2. Heterogeneity Perspective

#### 3.2.1. Heterogeneity Study of Family Firms

Heterogeneity is originally an important concept in the field of ecological research, which refers to “the complexity or variability of system attributes in space or time” [74]. Daspitetal (2021) introduced it to the study of family firms, and defined family firm heterogeneity as the degree of difference between family firms in category allocation and changes [75]. Although the differences within family firms are much greater than those between them and non-family firms [76], most scholars subconsciously set family firms against non-family firms, leading to the mainstream of the research field to depict the difference between the two [75]. The literature review revealed that the heterogeneity of family firms mainly includes: “the heterogeneity of personnel involved”, “the heterogeneity of business objectives”, “the heterogeneity of governance”, “the heterogeneity of firm resources”, and “the heterogeneity of the output of family firms” [17,77,78]. The research on the social responsibility behavior of family firms took the lead from the perspective of heterogeneity, such as the participation of the founder [79], intergenerational ownership [80], family values [81], and personal characteristics of managers [82], etc. However, studies targeting the environmental behavior of family firms from a heterogeneous perspective are not common and have only started to emerge from recent years.

Based on the homogeneity of family firms, different conclusions have been drawn on their behavior, which suggests that we can carry out the heterogeneity study of family firms in addition to the comparative study of family firms and non-family firms. Since the family characteristics of different family firms are different, and the industry, scale and system may affect their environmental behavior to varying degrees, observing these more direct and specific factors will help us better explore the motivation of family firm environmental behavior.

#### 3.2.2. Heterogeneity Study of Environmental Behavior of Family Firms

In studies on the environmental behavior of family firms from a heterogeneous perspective, family ownership, family shareholding, family control, family stewardship, and innovation management have been key considerations [83,84,85]. Differences among family firms occur as a result of family and founder involvement in the family firm. Changes in the values and characteristics of the founder and the next generation, as well as changes in family ownership, structure, and nonfinancial goals, are the main antecedents of family firm heterogeneity, influencing their environmental decisions and environmental behavior accordingly [83]. Some scholars have also taken family involvement time into consideration and found that there is an inverted U-shaped relationship between family involvement time and corporate environmental responsibility [86,87]. The degree of family involvement in corporate governance and its optimal governance configuration can drive the environmental social performance of family firms, and the combination of 100% family ownership or the presence of high family in the board and the participation of low family in management is the optimal combination of family participation in corporate governance, which is conducive to the adoption of pro-environment behavior by enterprises [56].

Studies from different countries and different industries have also provided new perspectives to examine the environmental behavior of family firms from a heterogeneous perspective, such as Kazancoglu et al. (2021) who used a system dynamics model to study cleaner production in family firms found that ethical development and innovation management were the main influences on the establishment of socially responsible and environmental management systems in firms [85]. Nikolakis et al. (2022) conducted a targeted study of family firms in Chile and India and found that conflict was the main cause of reduced ESS (environmental and social strategies) in family firms, but higher trust generated less rational conflict, which favored ESS; and family firms with higher SEW in India were more likely to adopt ESS [51]. It is noteworthy that compared with many studies on family firms and non-family firms, there are few studies on family firm environmental behavior from the perspective of heterogeneity.

The theory of social emotional wealth with family firms as the research object marks the exploration of the unique theory of family firms and lays a theoretical foundation for the analysis of family firms’ environmental behavior [18,22]. In terms of research methods, empirical studies and case studies remain the dominant form of environmental behavior research in family firms, while all measures concerning socioemotional wealth use alternative measures [83]. It is noteworthy that gradually some scholars are trying to draw on research methods from other fields to interpret family firms. In addition, the application of qualitative comparative analysis in business management research provided a worthwhile new way to study the heterogeneity of family firms in terms of methodology [88].

## 4. Study on the Influence Effect of Environmental Behavior of Family Firms

The study of the influence effect of corporate environmental behavior is from the perspective of the firm itself, emphasizing how the firm can transform its living environment to achieve high performance. From the existing studies, scholars have mainly explored the influence effect of corporate environmental behavior of family firms from the following three aspects.

### 4.1. Interorganizational and Intraorganizational Relationships of Family Firms

In terms of the level of influence effect of family firm’s environmental behavior, studies can be divided into three levels: interorganizational, organizational, and intraorganizational. From the interorganizational level, Cordeiro (2021) tested the impact of family firms’ environmental behavior on financial markets based on the first “green ranking” of the top 500 U.S. companies by Newsweek in 2009 and found that shareholders had a significantly more positive response to companies with higher environmental performance rating [23]. This effect is significantly more positive and stronger in family firms. Different from the “second type” agency conflict between most family owners and minority shareholders, large shareholders may not necessarily occupy the rents of small shareholders in investment environment activities, while positive environmental behavior of family firms can bring positive returns to firms in the stock market [56]. The goals related to social emotional wealth make family firms more willing and motivated to participate in inter-firm R&D collaboration and innovation, which enables them to build more effective R&D partnership interactions to develop higher value green innovations [89,90].

At the organizational level, existing studies have found that family firms’ environmental behavior can bring positive performance in terms of legitimacy [91], competitiveness, and corporate reputation [7], as well as improve corporate financial performance. Family firms have a stronger environmental orientation than non-family firms, and the link between environmental management activities and innovation is stronger in family firms, explained by Ozsomer et al. (1997), in terms of higher organizational flexibility in family firms [92]. Some studies also believe that environmental behaviors of family firms, such as pollution prevention and control, environmental management, and sustainable development strategies, have a positive impact on the construction of competitive advantages and brand equity cultivation [37,72,93].

In terms of intraorganizational influence effects, for family owners with a long-term orientation, the firm is often seen as a vehicle for nurturing the family’s future and achieving continuity that can provide jobs, security, and income for the next generation. In family firms, the implementation of sustainability strategies can enable family members to have sustainable economic resources [94]. For the general employees of family firms, corporate environmental and social strategies can generate more positive attitudes among employees to deal with environmental issues and thus be more supportive of corporate environmental and social strategies, as confirmed in Nikolakis’ (2021) investigation of corporate environmental and social strategies in India and Chile [51]. That is, respondents in firms with a sustainability vision expressed more positive environmental attitudes and had lower perceptions of intra-firm conflict, suggesting that employees may comply with environmental regulations in order to avoid internal conflicts.

### 4.2. Economic Performance, Institutional Performance, and Environmental Performance

From the perspective of the effects of environmental behavior of family firms, environmental behavior of family firms can bring economic, institutional and environmental performance, which is consistent with Bansal and Roth’s (2000) study of firms’ motivations for responding to ecological requirements [95], namely competitiveness, legitimacy, and ecological responsibility. Competitiveness refers to the competitive advantage gained through environmental behavior, legitimacy refers to the implementation of environmental behavior by firms to meet the requirements of laws and regulations, and ecological responsibility is a motivation to satisfy a wide range of social interests (see Table 3).

#### 4.2.1. Economic Performance

The direct effect of environmental behavior of family firms on economic performance is mainly reflected in financial performance, and the improvement of financial performance is mainly achieved through environmental management and green innovation. In fact, the availability and accessibility of financial and human resources is a prerequisite for firms to excel in the environment [96]. Craig (2006) surveyed for-profit firms in all industries in one western state of the United States, where respondents were asked to rate the positivity of their firm’s involvement in environmental activities, and found that family firms had more positive attitudes toward natural environmental policies than non-family firms and that natural environmental policies had a stronger positive effect on firm innovation and financial performance [97]. Tan (2021) studied the impact of environmental information disclosure on the level of bank loans and the moderating role of executive characteristics in it, using family firms listed in the A-share manufacturing industry in Shanghai Stock Exchange of China from 2016 to 2018, and found that positive environmental information disclosure by family firms can expand the size of corporate loans, thus providing support for corporate financing and improving corporate financial performance, while the financial background of managers has a positive moderating effect on the environmental information disclosure of family firms’ and the scale of bank loans [98].

Compared with general enterprises, family firms have a preference for non-economic motive goals [18], and family firms’ environmental behavior can bring some potential economic performance to the firm, which is mostly presented in the form of non-economic performance [32,56,99]. Based on the driving factors of green innovation strategy of family firms, it is found that family inheritance intention can promote the implementation of green innovation of family firms, while family control intention will inhibit the green innovation intention of family firms [48]. Family firms’ long-term orientation theory, on the other hand, argues that family firms place more importance on the long-term sustainability of the firm than on short-term profits, and therefore family firms value environmental investments in an attempt to pass on a healthy business to future generations [100]. Without sacrificing the interests of other stakeholders in exchange for family benefits [83], long-term orientation has a positive impact on sustainable corporate behavior, while sustainable behavior enhances the long-term orientation of family firms [3].

#### 4.2.2. Institutional Performance

The reflection of environmental behavior of family enterprises in institutional performance mainly focuses on the pressure and response of family firms to environmental regulation. Berrone (2010) argued that family owners are more susceptible to negative external evaluations [22], value social legitimacy itself, and therefore tend to engage in substantive environmental practices rather than superficial compliance when faced with environmental institutional pressures. This conclusion is confirmed by comparing the environmental performance of family and non-family listed firms. Family and non-family firms do not react the same way when faced with institutional pressure and institutional support; family firms are more inclined to comply with environmental regulatory pressure and implement pollution prevention strategies, but the crowding-out effect of institutional support may lead family firms to reduce their pollution prevention investments. It suggests that in the face of institutional pressure, family firms are more concerned with their own legitimacy, and implementing environmental practices can help family firms avoid the losses caused by the violation of environmental regulations, including fines and damage to corporate reputation. While in the face of institutional support, family firms are more concerned about whether their pollution prevention investments can bring additional benefits, and implementing environmental behaviors outside of regulations may not bring economic benefits to the firm [24].

#### 4.2.3. Environmental Performance

The impact of family involvement on their environmental performance is multidimensional, and different research dimensions may lead to different conclusions. Family shareholding can make families more concerned about short-term losses financially than investing in environmental governance, however, as family control time increases, families also improve environmental performance for the sake of socioemotional wealth such as children’s legacy and image in the community, but the role of family control time is only more significant when the family has absolute control [101]. Measuring the environmental social performance of family firms in four dimensions: compliance with environmental regulations, limiting environmental impacts beyond compliance, preventing and mitigating environmental crises, and educating employees and the public about the environment, it was found that different levels of family involvement in ownership and management, as well as the composition of the board of directors into configurations, improved the environmental and social performance of family firms and the enthusiasm to participate in social responsibility [56]. When family firms are highly embedded in society, family firms tend to be more concerned about environmental performance than non-family firms due to the protection of SEW [69].

In terms of green innovation capacity development, the risk-averse tendency of family firms and the insufficient introduction of talents in family firms lead to the lack of green innovation capacity [102]. Environmental innovation, rather than activities to prevent environmental problems, are more representative of positive corporate initiatives to face environmental problems. Family involvement is more likely to promote environmental innovation in firms [55,67], and there is a positive relationship between family involvement and the value of green innovation [103,104]. As the research progressed, more other factors are creatively included in the study. For example, family members’ identity with the firm has a significant and positive overall impact on sustainable products and processes, and firm competence has a significant positive impact on environmental performance [99].

Different answers have also been given by scholars as to whether family firms have better environmental performance. Based on the meta-analysis of the literature on the environmental performance of family firms from the perspective of agency theory, it is found that family involvement has a negative impact on the environmental performance of the firms, mainly due to the costly agency costs resulting from family members seeking private benefits from the firm [64]. In terms of environmental information disclosure, family firms with family control and influence as the main dimension of SEW tend to reduce environmental information disclosure, because the firm’s proprietary information contained in environmental disclosure, as well as the increased corporate visibility generated by information disclosure, will threaten the family’s control power and influence on the firm [38].

### 4.3. Environmental Behavior of Family Firms and Public Health

At the beginning, public health mainly focused on the living environment, clean drinking water and sewage treatment. Later, public health gradually involved a wide range of fields, but all related to the environment and sanitation conditions. The environment has a fundamental influence on public health and determines the state of human existence. If the public health wants to maintain a good state of development for a long time, we must pay attention to the maintenance and management of environment. Enterprises are the main body of pollutant discharge, and the key link in environmental governance, and enterprises are also one of the main bodies that affect public health. However, there is not much literature on the impact of environmental behavior on public health, especially in family firms, and it is mainly focused on environmental responsibility, sustainable development, and employee health.

#### 4.3.1. Environmental Pollution, Environmental Responsibility, and Public Health

The existing literatures have strongly confirmed that environmental pollution is detrimental to public health. For example, a study by Chen et al. (2013) concluded that heating policies based on the Qinling-Huaihe River divide in China increased the total pollution in northern China and shortened the life expectancy of northern residents [105]. Cheung et al. (2020) constructed instrumental variables based on wind direction and air pollution in the surrounding area and found that air pollution increases residential mortality [106]. Most of the traditional economic theories treat the environment as a pure public good, and thus firms are reluctant to increase their investment in environmental protection in the absence of other incentives. As a result, much of the policy advice has focused on how the government can monitor firms and strictly control the pollution and damage caused by their production to the environment. This way of thinking generally assumes that firms are passive in areas such as environmental protection and have little incentive to be proactive in protecting the environment, reducing pollution, and maintaining employee health. However, with the development of human capital theory and health economics, the limitations of the original research have been theoretically recognized and it has been found that companies are not completely unmotivated to invest in environmental protection. Unfortunately, few research has yet focused on the exact impact of corporate environmental behavior on public health.

#### 4.3.2. Healthy Capital, Employee Health, and Public Health

Any issue related to public health can be understood as a public health issue, such as environmental protection and health education. Then, from the perspective of the study of corporate environmental behavior, the promotion of employee health is also a component of public health. Modern human capital theory suggests that a worker’s stock of human capital consists primarily of elements such as health, knowledge, skills, and work experience, all of which enhance individual productivity. Each person acquires an initial stock of health capital through heredity, which depreciates with age, but can also increase due to health investments, and the health investments of employees in a company are directly related to the company’s environmental protection expenditures. This health capital, together with the efficiency wage provided to employees, becomes the two sources of improving employee productivity and corporate profits [107].

It has also been found that family firms may be more reluctant to fulfill their employee safety and health responsibilities than non-family firms, but as they operate for longer periods of time, family firms begin to pay more attention to employee health and safety, which may be explained by the fact that in the early years of operation, family firms tend to focus on accumulating capital and therefore pay more attention to their business performance, and as the business situation reaches stability, family firms will more substantially responsible [108]. Kariyapperuma and Collins (2021) found that the founder’s personal values and family values, as well as the health and safety requirements of family members, non-family employees and customers, play a leading role in the environmental behavior of family firms when analyzing the environmental information of family firms operating in the New Zealand wine industry [83]. At the same time, some family firms have shown a desire to create a healthy living environment through environmental behaviors to ensure the health of their family members, as well as their employees [83]. In addition, some studies have also found that family firms perform better in focusing on employees’ mental health and occupational safety and health [109].

## 5. Conclusions and Discussion

In recent years, family firm research has made great theoretical progress, revealing the inter-embeddedness between family and business as two systems and their complexity in organization and management, which goes beyond the scope and significance of the existence of business as a pure economic organization in the past. Family firm research is becoming one of the most important and promising fields of management practice theory innovation, attracting more and more attention from management, economics, sociology, and other disciplines. Family firms’ environmental behavior is an important theoretical branch of family firm research, which is of great significance to enrich and deepen the theoretical and practical research on family firms.

First, the research on environmental behavior of family firms involves many subjects and the hierarchical relationship is very complicated. However, according to existing studies, there is no significant difference between family and non-family firms in terms of environment-related activities, green innovation, and performance in the long term. Most environmental behavior models generally assume that enterprises participate in a series of homogeneous environmental actions [110]. However, only by properly considering different types of firms (family firms and non-family firms) and different dimensions of environmental behavior can we more accurately understand the overall view of corporate environmental behavior.

Second, studies on the factors influencing the environmental behavior of family firms from a homogeneity perspective have mostly focused on revealing the overall impact of family firms on environment, or specific characteristics such as long-term orientation and cross-generational succession on the environmental behavior of family firms. Since family firms are regarded as homogeneous and differences among family firms are not considered, different scholars have reached many different or even opposite conclusions. Although scholars have been able to explain these conclusions based on certain theoretical perspectives (e.g., SEW theory), they have not yet contributed enough to practical insights such as how to optimize the environmental behavior of family firms or improve the environmental performance of family firms.

Third, the study of factors influencing the environmental behavior of family firms from a heterogeneous perspective has only started to emerge in recent years, but the related research is very creative and practical. In contrast to the previous binary comparison between family firms and non-family firms, examining the factors influencing the environmental behavior of family firms from a heterogeneous perspective provides an excellent research perspective and policy reference value for improving the environmental behavior of family firms. In fact, there are great differences among family firms. For example, different family firms show different forms of “family involvement”, and family firms in different life cycles also show different environmental behaviors. However, the research on the factors influencing the environmental behavior of family firms from the perspective of heterogeneity has not been systematized, and none of the underlying theories have formed a mainstream trend either. The research samples are often scattered and may have high reference value for specific countries or regions, but no scholars have used many global samples to conduct comprehensive research. In general, there are still many gaps in the research on the heterogeneity of corporate environmental behavior.

Fourth, the mechanism of the influence effect of corporate environmental behavior is very complex, so there are many disagreements on the influence effect of family firms’ environmental behavior. Most studies on the economic performance, environmental performance, and institutional performance of family firms found that there was no significant difference between family firms and non-family firms, but the specific influencing factors were not the same [71]. This may be because most studies have focused on the comparison of family and non-family firms and ignored the heterogeneity among family firms, such as not breaking down the different family control styles. Although scholars affirm the important role of socioemotional wealth in family firm decision-making, there are significant differences in the ability and willingness of different family firms to achieve socioemotional wealth protection. As a combination of family and business, family firms often have multiple goals, which makes them base their decisions not only on “family logic”, such as maintaining family reputation and caring for family honor, but also on “business logic”, such as considering corporate profits (see Table 4).

Fifth, there are no substantive implications for simply arguing whether family firms’ environmental behavior can promote performance improvement and environmental health. Which environmental behaviors can promote enterprise performance and benefit environmental health and public health under what circumstances have more practical and policy implications. Unfortunately, there is a lack of research on the mechanisms of the various influence effects of environmental behaviors, which cannot effectively explain why sometimes the same environmental behavior has different institutional, economic, and environmental effects. Currently, scholars are unable to provide effective measures for companies to adopt environmental behaviors that can promote both corporate performance and ecological improvement, thus making it difficult to provide meaningful insights for governments to formulate corresponding environmental behavior incentives and regulation policies. Therefore, future research should also focus on the mechanism of environmental behavior influence effects of family firms.

## 6. Implications and Future Research Direction

### 6.1. Implications

It can be found from the literature review that the internal and external factors such as family involvement degree, environmental regulation, and sustainable development all affect the environmental behavior of family firms to varying degrees. In the face of environmental pollution, family firms are not only the negative responders to environmental pressure, but also the positive promoters of environmental protection and green production innovation. For family firms themselves to become active promoters of environmental behaviour, they first need a modern transformation in their governance structure, such as bringing in more external directors and professional managers with green professional knowledge, technology, and experience. Secondly, family firms need to arrange in advance the selection and training of the second generation of the family, select suitable successors and consciously cultivate their family spirit awareness, innovation, and environmental awareness, which is an important guarantee for the effective implementation of green responsibilities of family firms. From the perspective of external pressure, family firms are mainly faced with the pressure from external stakeholders on the protection of natural environment and the greening of production methods, such as government environmental regulations. Therefore, family firms can be promoted to become the implementers and promoters of environmental protection and green innovation from the aspects of policy formulation and creating a good competitive environment.

### 6.2. Future Research Direction

From the existing research results, the research on the factors influencing the environmental behavior of family firms is still at the stage of strife. There is a lack of in-depth and systematic research on the factors affecting the change of specific environmental behaviors of family firms and their interactive mechanisms, and there is also a lack of research on the antecedents of the factors influencing the corporate environmental behavior. In the future, multiple theories can be integrated to open the black box of the mechanism of environmental behavior of family firms from multiple perspectives and in all aspects. The measurement indexes of the influence effect of family firms’ environmental behavior are not perfect, and the influence effect and generation mechanism of family firms’ environmental behavior also need to be studied. Future studies may consider the following aspects to analyze the generation mechanism of environmental behavior effects: Variables such as the size of family firms, the industry they belong to, the time of implementation of environmental behaviors, and intergenerational inheritance are included in the theoretical model to reveal the moderating effects of these variables on the environmental behaviors of family firms and their influence effects and further explore the relationship and interaction of the influence effects of various environmental behaviors. Therefore, it can provide a reference for the government to formulate targeted policies to encourage and regulate the environmental behaviors of family firms and guide enterprises to adopt environmental behaviors that can not only enhance the enterprise value and promote the sustainable development of enterprises but also promote the improvement of ecological environment [111].

Although many empirical studies have attempted to understand the environmental performance of family firms around the world, most of the studies collect data by doing questionnaire surveys and the measurement indicators of environmental performance do not clearly define “environmental intention” and “actual environmental performance”. Therefore, the direction and degree of impact of family participation on environmental protection are not clear. This brings great challenges to the theoretical development of a family firm theory and environmental management model and also points out future research directions [28,55]—that is, to clearly distinguish the environmental concerns and environmental performance of family enterprises and further study the ability of family firms to transform environmental achievements. In addition, considering that the environmental behavior of family firms is largely affected by their family characteristics, the driving factors of a family firm environmental performance should not only be discussed in the descriptive and static ways. In addition to researching the dynamic and long-term dimensions, it will also be very interesting to further study some other aspects that can affect family intentions, such as family values, specific customs and rituals, and family religious beliefs.

A literature review of the environmental behavior of family firms reveals that there is no theory that comprehensively explains the influencing factors and influence effects of family firms’ environmental behavior. When it is difficult to explain the nature of family firms with a particular theory, a feasible approach and idea is to apply or integrate multiple theories simultaneously for complementary explanations [6]. For example, the theories of family firms are integrated with existing classical theories of environmental economics and management to explain important academic issues in the field of environmental behavior of family firms, especially cross-cutting ones. The idea of theoretical integration helps to promote the value of the application of several classical theories or even to propose new theoretical frameworks, on the one hand, and to understand more deeply the driving factors of the environmental behavior of family firms on the other. The complexity of family firms’ problems and the characteristics of multi-actor interactions determine the multiple influencing factors of family firms’ environmental behavior overlapping each other, then theory integration becomes an important direction for the innovation and development of family firms’ environmental behavior theories.

With the rapid development of digital technology, the necessity and urgency of digital transformation of family firms are further highlighted, which will profoundly affect corporate decision-making and environmental behavior. Digital transformation is well positioned to meet the environmental sustainability needs required by the industry, and leading companies are using digital technologies to turn environmental challenges into significant business opportunities. Studies have begun to focus on how family firms can achieve digital transformation through intergenerational transmission in the digital process [112]. There are also studies that explain how digital technology and digital transformation positively affect corporate environmental behavior, such as promoting proactive environmental strategies [113], improving of energy efficiency and resource management [114], building digital supply chain platforms [6], increasing green technology innovation [115], and alleviating financing constraints and attract government subsidies [116]. The magnitude of the effect of digital technology on corporate environmental performance is also influenced by corporate heterogeneity factors such as board structure and educational background [117], dimensions of digital finance [118], and so on. Although the digital age has not given stakeholders greater influence on corporate environmental behavior [119], the influence of the family behind the firm remains to be further investigated. In this context, how resource heterogeneity affects the digital transformation of family firms [120], the mechanism of the role of heterogeneous characteristics of firms on their digital technology adoption and digital transformation, and the impact of digital transformation of family firms on their environmental behavior need to be further investigated.

It should be pointed out that the research on the environmental behavior of family firms in this paper is limited to literature summaries, presentations and brief reviews, and the different research methods, variable selections and model constructions of different literature are only listed in Table 4. There is no further comparative study of different research conclusions to further explore the environmental behavior of family firms, which will become our next research content.

## Figures and Tables

**Table 1:** Comparative study on the environmental behavior of family enterprises in different dimensions.

Research Dimension	Environmental Responsibility	Economics	Strategic Management
Research level	Organization	Industry, organization	Organization
Research topic	Strengthen the environmental responsibility of enterprises from the aspect of ethic	The impact of environmental regulation on the competitiveness of family firms	Family firm environment behavior choice and the source of competitive advantage
Research method	Qualitative research	Model construction and metrological inspection	Qualitative research

**Table 2:** The lineage of research on the factors influencing the environmental behavior of family firms.

Research Classification		Representative Views
Homogeneity Perspective Study	SEW Theory	Family-controlled public companies will protect their socioemotional wealth by acquiring better environmental behavior than non-family firms. (Berrone et al. 2010)
		Under the combined effect of SEW goals and economic wealth goals, the environmental behavior of family firms initially lags behind that of non-family firms, but gradually catches up to form a convergence process. (Doluca et al. 2018)
		Family firms have a stronger propensity to green innovation than non-family firms driven by the internal motivation to preserve extended social emotional wealth and external institutional pressure. (Ma, Zhu and He 2020)
	SEW Dark Side Theory	There is a negative effect of family ownership and management involvement on the fulfillment of ER of private enterprises. (Zhou and Zhao 2017)
		Absolute family ownership of firms may reduce incentives and limit opportunities for family firms to improve their environmental social performance. (Samara et al. 2018)
		Family firms with the most significant family control and influence on the SEW dimension provide less environmental information than non-family firms. (Arena and Michelon 2018)
	Agency Theory	Family firms do not perform as well as non-family firms in terms of environmental performance, and investment in training explains the negative association between family majority shareholders and environmental performance. (Dal Maso 2020)
		Majority shareholders do not necessarily incur a loss of benefits to minority shareholders when investing in environmental activities, and type II agency problems do not arise in the environmental behavior of family firms. (Cordeiro et al. 2021)
		Family firms are more responsible to shareholders than non-family firms when it comes to making environmental investments. (Abeysekera and Fernando 2020)
Heterogeneity Perspective Study		Family ownership, family shareholding, and family control ability influence the environmental behavior of family firms. (Kariyapperuma and Collins 2021; Zhou and Zhao 2017; Zhu et al. 2022; Zhou et al. 2020)
		Family members as chairman, family management rights, innovative management, and family willingness to control influence the environmental behavior of family firms. (Kazancoglu et al. 2021; Benjiang Ma et al. 2022; Zhu, Lina et al. 2022)
		The personal and family values of the founder and the health and safety requirements of family members play a dominant role in the environmental behavior of family firms. (Kariyapperuma and Collins 2021)

Source: Based on the relevant literature.

**Table 3:** Main viewpoints on the influential effect of family firm environmental behavior.

Influence Effect	Main Viewpoints	
Institutional performance	Corporate environmental legality (Berrone et al. 2010; Fan et al. 2021)	
Economic performance	Financial performance	Positive equity market returns (Cordeiro et al. 2021) Improvement in the level of bank lending (Tan 2021) Improvement in financial performance and operating performance (Dangelico et al. 2019) Improvement in innovation capacity and financial performance (Craig and Dibrell 2006)
	Nonfinancial performance	Improvement of corporate reputation (Dangelico et al. 2019) The protection of socioemotional wealth (Berrone et al. 2010; Cruz et al. 2014; Samara et al. 2017; Dayan et al. 2019) The protection of extended socioemotional wealth (Ma et al. 2020) Meeting the long-term orientation needs of businesses (Breton-Miller et al. 2016) Holding the future economic income of families (Miller et al. 2009) Satisfying internal stakeholders (Huang et al. 2009) Ensuring product quality, employee health and corporate reputation (Kariyapperuma et al. 2021)
Environmental performance	Positive correlation	Preventing and mitigating environmental crises and reducing environmental impacts (Samara et al. 2018) Development of green innovation capacity (Han et al. 2022) Positive environmental attitudes of employees (Nikolakis et al. 2022) Environmental education for employees (Samara et al. 2017)
	Negative correlation	Threaten the family’s control and influence over the business (Arena and Michelon 2018) Burden the full economic risk of environmental investments (Dekker and Hasso 2016) Costly agency costs for family firms (Miroshnychenko et al. 2022) Inadequate capacity for green innovation and risk-averse tendencies (Aiello et al. 2021)

**Table 4:** Representative literature on the relationship between family firms and corporate environmental issues (excerpt).

Researcher	Theory	Independent Variables	Dependent Variable(s)	Sample	Relationship
Craig and Dibrell (2006)	Organization and natural environment literature	Family firms, natural environmental policies	Natural environmental policies, firm’s innovation, and firm’s performance	391 small and medium U.S. firms	+/−
Berrone et al. (2010)	Socioemotional wealth perspective	Family firms (dummy variable), local roots, CEO stock ownership, family CEO and CEO duality	Environmental performance (human toxicity potential)	194 U.S. firms required to report their emissions to the EPA	+/−
Dekker and Hasso (2016)	Institutional theory	Family firms, social embeddedness	Environmental performance focus based on the Business	1452 private Australian small and medium-sized enterprises	−/+
Samara et al. (2018)	Socioemotional wealth perspective	Family ownership, family involvement in management	Environmental social performance of the STEP	146 family firms of the STEP database	+/+/0
Dangelico, Nastasi, and Pisa (2019)	Multiple case study	Family firms vs. nonfamily firms	Green innovation	14 small enterprises from the agri-food industry in Italy (7 family and 7 nonfamily firm	0
Dal Maso et al. (2020)	Secondary agency theory	Family ownership and investments in training and development practices (mediation)	Asset4 environmental performance score (environment)	3901 firm-year observations from 2002 to 2016 distributed across 56 countries	-
Fan (2021)	Agency theory and institutional theory	Family ownership, founder-CEOs	pollution prevention strategy	2348 Chinese private enterprises in primary and secondary sectors.	−/+
Nikolakis (2022)	Socioemotional wealth perspective (using a stated choice method)	Relational conflict, trust, sustainability vision, SEW, family in control, environmental norms	Environmental and social strategies	119 family business representatives from Chile and 120 from India	−/+

## Data Availability

Not applicable.
